# Functional Status in Older Adults with Peripheral Artery Disease in Korea: A Cross-Sectional Study

**DOI:** 10.1093/geroni/igab046.3494

**Published:** 2021-12-17

**Authors:** Yesol Kim, Mihui Kim, Gi Wook Ryu, Mona Choi

**Affiliations:** 1 Yonsei University, Seoul, Seoul-t'ukpyolsi, Republic of Korea; 2 Namseoul University, Cheonan, Ch'ungch'ong-namdo, Republic of Korea

## Abstract

Peripheral artery disease (PAD) is a chronic, progressive atherosclerotic disease resulting in worse functional status. It is an important factor that affects mobility and quality of life in older adults with PAD. This study aimed to identify the functional status and its associated factors of older adults diagnosed with PAD. We conducted a cross-sectional study among older adults aged 65 above diagnosed with PAD at a tertiary hospital in Seoul, Korea. Participants' functional status was measured using a Walking Impairment Questionnaire (WIQ) which consisted of distance, speed, and stair-climbing. We measured cardiac health behavior, social support, health perception, and clinical manifestation through self-administered questionnaires. Among 94 participants, the mean age was 74.98±6.21 years, and 91.5% were male. The mean score of WIQ was 0.59±0.30 out of 1; the mean scores for distance, speed, and stair-climbing of WIQ were 0.67±0.40, 0.45±0.27, and 0.64±0.37, respectively. Participants' functional status was significantly associated with age (β=-0.012, P=.002), sex (β=-0.284, P=.001), ulcers of the lower extremity (LE) (β=-0.242, P=.031), using a walking-assist device (β=-0.240, P=.002), walking difficulty due to pain of LE (β=-0.142, P=.006), and health behavior about physical activity (β=0.099, P=.021). This regression model predicted 53.5% of participants' functional status (F=8.63, P<.001). This study indicated that younger age, female, independent walking, no ulcers of LE, no walking difficulty, and higher physical activity behavior were significantly associated with better functional status in older adults with PAD. Therefore, healthcare professionals should develop and provide interventions to promote physical activity and alleviate symptoms to enhance functional status.

